# Brugada syndrome precipitated by uncomplicated malaria treated with dihydroartemisinin piperaquine: a case report

**DOI:** 10.1186/s12936-024-05099-3

**Published:** 2024-09-17

**Authors:** Muzakkir Amir, Irmayanti Mukhtar, Pendrik Tandean, Muhammad Zaki Rahmani

**Affiliations:** 1 Department of Cardiology and Vascular Medicine, Medical Faculty, Hasanuddin University, Makassar, Indonesia; 2 Dr. Wahidin Sudirohusodo National General Hospital, Makassar, Indonesia; 3 Medical Faculty, Hasanuddin University, Makassar, Indonesia

**Keywords:** Brugada syndrome, Arrhythmia, Malaria, Antimalarial drug, Heart disease, Electrocardiogram

## Abstract

**Background:**

Cardiovascular events following anti-malarial treatment are reported infrequently; only a few studies have reported adverse outcomes. This case presentation emphasizes cardiological assessment of Brugada syndrome, presenting as life-threatening arrhythmia during anti-malarial treatment. Without screening and untreated, this disease may lead to sudden cardiac death.

**Case presentation:**

This is a case of 23-year-old male who initially presented with palpitations followed by syncope and shortness of breath with a history of malaria. He had switched treatment from quinine to dihydroartemisinin-piperaquine (DHP). Further investigations revealed the ST elevation electrocardiogram pattern typical of Brugada syndrome, confirmed with flecainide challenge test*.* Subsequently, anti-malarial treatment was stopped and an Implantable Cardioverter Defibrillator (ICD) was inserted.

**Conclusions:**

Another possible cause of arrhythmic events happened following anti-malarial consumption. This case highlights the possibility of proarrhytmogenic mechanism of malaria infection and anti-malarial drug resulting in typical manifestations of Brugada syndrome.

## Background

Brugada syndrome (BrS) is a major cause of cardiac arrest and sudden death in young people with normal heart structure. Arrhythmic manifestations and cardiac arrest in BrS tend to occur in men and populations in Southeast Asian countries [1, 2]. Clinical manifestations of BrS can vary from mild discomfort in the form of syncope to sudden death. It is an autosomal dominant disorder characterized by ST elevation and negative T waves in right heart electrocardiography (ECG) leads without structural cardiac abnormalities, which occur because of mutations in the cardiac sodium gene SCN5A resulting in loss of cardiac sodium channel function, decreased sodium influx, and depolarization phase shortening of the action potential, which is also associated with sudden cardiac death [3]. BrS is extremely rare in Southeast Asian and East Asian countries, this may be due to poor reporting or diagnosis resulting from facility limitations, meanwhile Indonesia is endemic for malaria [2, 4]. Studies have highlighted the cardiotoxic effects of anti-malarial drugs, particularly piperaquine that has been linked to QT prolongation and sudden cardiac death, emphasizing the need to review their cardiovascular safety profiles [5–7]. Very few studies have evaluated the association between anti-malarials and cardiovascular adverse events.

## Case presentation

A 23-year-old male came to the emergency unit of Dr. Wahidin General Hospital complaining of palpitations. He described episodes that had occurred approximately 4–5 times in the last 4 months. There was a history of fainting, which was previously preceded by palpitations.

**Fig. 1:**
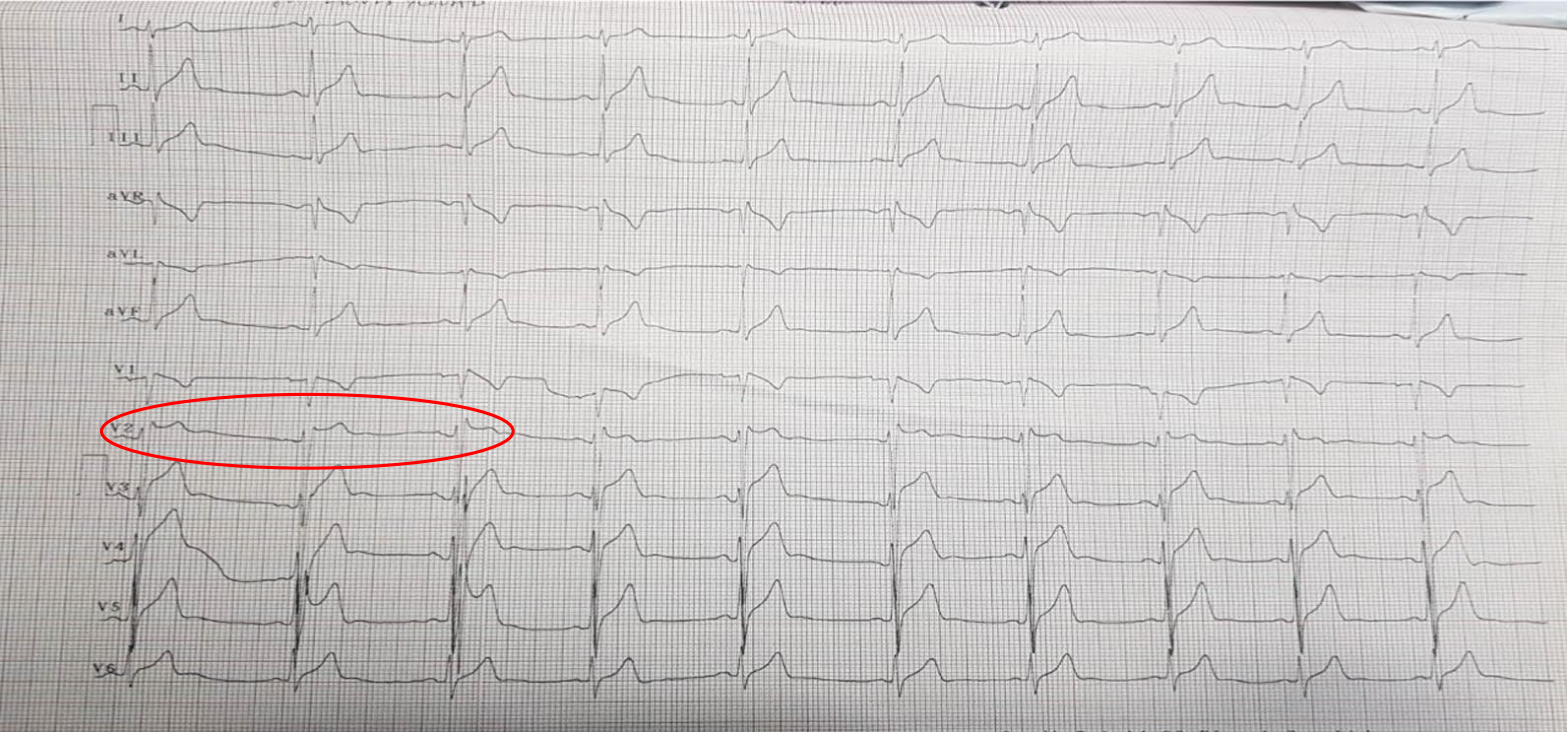
Baseline ECG shows Type 2 Brugada Pattern in V2 lead

**Fig. 2:**
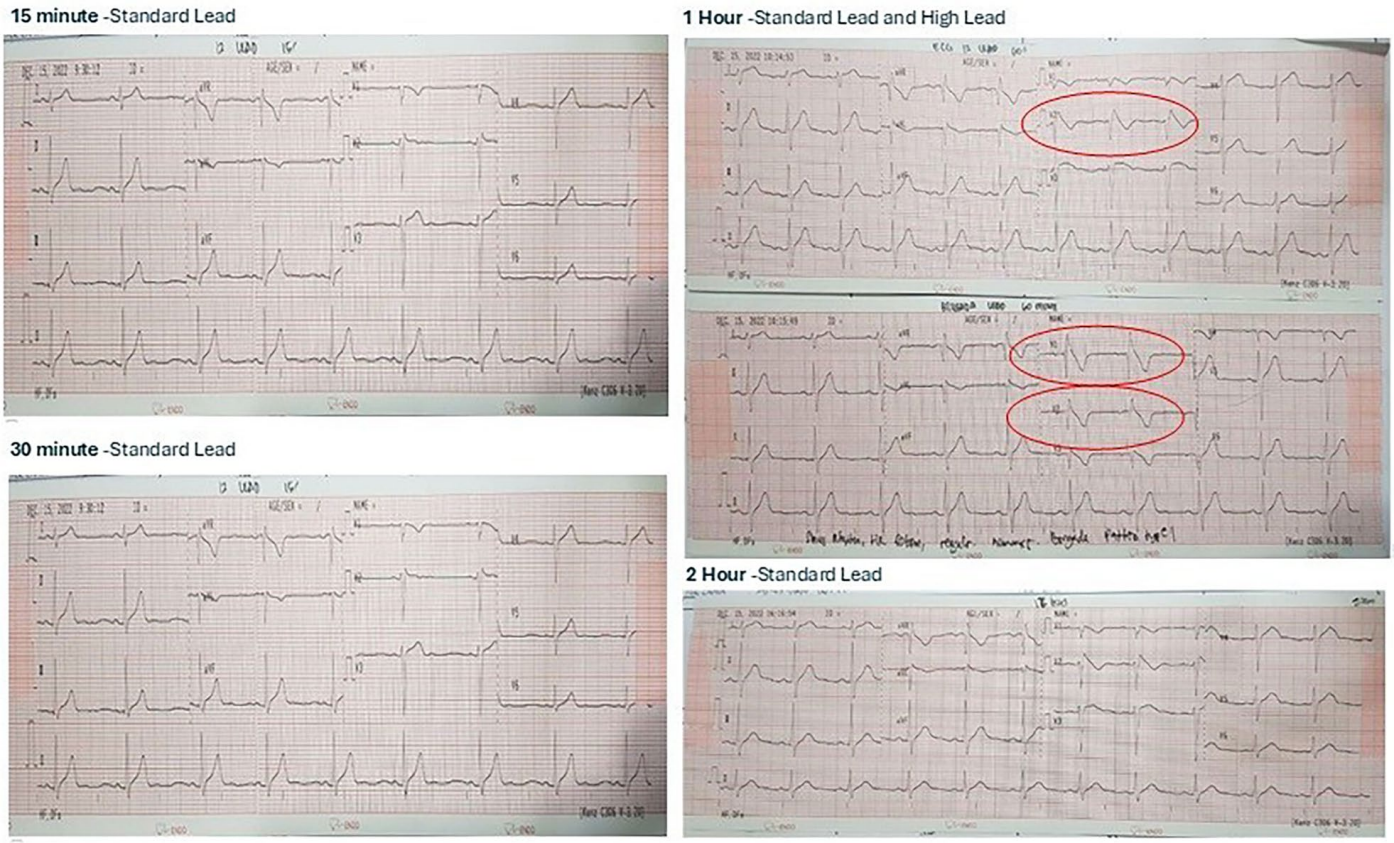
Flecainide Test Result shows Brugada Type 1 Pattern

He described atypical chest pain, like being stabbed during the palpitations. There was also a history of fever accompanied by chills. The patient had no family history of the same complaint. The patient had contracted malaria while on duty in Papua 1 year previously and was given quinine therapy. However, the patient subsequently experienced palpitations and tinnitus, so the treatment was stopped, and the patient was switched to DHP (dihydroartemisinin-piperaquine) for 3 days and primaquine for 14 days. The patient was hospitalized again for malaria 8 months later. Since then, the patient is self-medicating with DHP whenever he has fever because it did not resolve after taking fever-reducing medication (Figs. 1 and 2).

During the examination, the patient’s heart rate was 64 beats/mins, his respiratory rate was 18 breaths/minute, and his temperature was 36.5 C. Blood pressure was 114/63 mmHg. The patient was neither anaemic nor icteric, JVP R+ 2 cmH2O. Auscultation sounds, including vesicular sounds, rhonchi and wheezing, were absent. Regular heart sounds with no murmur and no oedema were observed. ECG revealed sinus rhythm, a heart rate of 64 beats/minute, normoaxis, a P wave of 0.08 s, a PR interval of 0.16 s, a QRS complex of 0.08 s, a soft curved ST elevation in V1 < 1 mm, saddleback ST elevation in V2 ≥ 2.0 mm, T reversal in V1, a QT interval of 360 ms, and Brugada type 2 pattern in V2. An oral *flecainid challenge test* (300 mg) performed to confirm the diagnosis of the disease. The test result showed a positive result at the 60th mins, and an image of Brugada pattern type 1 was observed (coved type) in V2 with an ST elevation > 2 mm. The images of the coved type in V1 and V2 were clearer in the right precordial lead position.

Malaria was confirmed with *Standareagen* rapid diagnostic test (RDT). Complete blood count and electrolytes laboratory shows slight changes (Tables 1 and 2), meanwhile echocardiography and chest X-ray results were normal.

**Table 1:** Complete blood count test

Parameter	Result	Unit	References
Haemoglobin	12.3	gr/dl	12–16
Haematocrit	36.8	%	37–48
Erythrocyte	4.1	10^6/ul	4.2–5.4
Leukocyte	9.3	10^3/ul	4.0–10.0
Thrombocyte	146	10^3/ul	150–400
MCV	88	fL	80–100
MCHC	27.1	pg	26–34
MCH	34.4	%	32–36
Lymphocyte	29.7	%	20-48
Monocyte	4.8	%	2–8
Neutrophil	83.8	%	50–70
Basophil	0.1	%	0.4–1.0

**Table 2:** Biochemistry, blood glucose, renal and Liver function, electrolytes, coagulation test

Parameter	Results	Unit	References
Random blood glucose	84	G mg/dl	70–140
Ureum	39	%	15-40
Creatinine	0.88	mg/dl	0,5-1,5
SGOT/AST	54	u/l	5–40
SGPT/ALT	38	u/l	5–40
Electrolytes			
Sodium	137	mEq/L	135–145
Potassium	5.0	mEq/L	3.5–5.0
Calcium	1.1	mmol/L	1.1–1.4
pH	7.16		7.35–7.45
Coagulation test			
PT	12.2	Seconds	11.7–15.1
APTT	30	Seconds	28.6–42.2
INR	1.22	ISI	0.1–2.0

The drug treatment was initially stopped and the patient consented to perform ICD implantation. Following ICD implantation, the patient experienced improvement, with no more palpitations felt, and further ECG findings were normal. The patient was discharged from the hospital with no post-ICD events. The malaria treatment then restarted to complete the course.

## Discussion

Brugada syndrome can develop and be triggered by various conditions, such as infection or drugs [4]. The European Heart Association classified BrS into three subtypes, but only two were prevalent in Asia with type 1 coved subtype being more common than type 2 and mixed type 3, occurring more often in men aged < 40 years [8]. In this case, the patient was a 23-year old man working in Papua, a region endemic for malaria. According to Fadilah et al. *Plasmodium falciparum* and *Plasmodium vivax* coexist in Papua, and the annual parasite index (API) is greater for men than for women [9].

ECG features of BrS were diagnosed when a Brugada type 1 ECG pattern of ST segment elevation occurred ≥ 2 mm in at least one right ventricular lead (V1 or V2) spontaneously or after intravenous administration of a sodium ion channel blocker. ECG morphology type 1 is the only pattern that is diagnostically significant for BrS [10]. In this case, a type 2 Brugada type was obtained in baseline ECG. To confirm this hypothesis, a standard flecanaide provocation test was carried out, an effective alternative when ajmaline was unavailable. After the provocation test, the ECG showed a Brugada type 1 pattern, where the coved ST segment elevation in lead V2 was > 2 mm, followed by a T inversion both in standard ECG lead placement and in the right high lead after one hour [11, 12].

Anti-malarial treatments, particularly those involving quinoline drugs (like chloroquine and quinine) and artemisinin-based combination therapy (ACT), are associated with arrhythmogenic cardiotoxicity. However, the extent of QT prolongation varies across different drugs and treatment regimens with more pronounced QT prolongation with quinoline, especially in higher doses [5]. Several studies highlighted that not all quinoline antimalarials carry the same level of risk. For instance, chloroquine and quinine were associated with more significant QT prolongation compared to other related drugs. QT prolongation is a marker of delayed cardiac repolarization and is associated with an increased risk of developing a life-threatening arrhythmia, such as Torsades de Pointes. However, the incidence of serious arrhythmic events remained relatively low [5, 13]. Bayesian meta-analysis compared the risk of sudden unexplained death associated with DHP to other anti-malarial treatments and found no significant difference in the risk of sudden death between DP and other commonly used anti-malarial drugs [6].

Pathophysiological mechanisms related to CVD and malaria are not well understood. One possible underlying factor of this condition is an unbalanced proinflammatory cytokine response or erythrocyte sequestration accompanied by increased cytoadherence to the endothelium. Hence, cardiomyocytes modulate ion channel membranes to decrease Ito, Ikr, and Iks and upregulate ICaL and Ina, resulting in ICaL overload, which leads to prolongation of action potential. The patient presented to the hospital with routine anti-malarial consumption and afebrile conditions, we suspected that this medication could have masked or reduced the impact of inflammation on arrhythmic events and reduce fever [14, 15]. Although the patient did not exhibit febrile during the examination, it is possible that they had a previous occurrence of fever. Several studies reported the occurences of fever possibly become a modulating factor that unmasked BrS, so this possibility cannot be ruled out because fever can precipitate Brugada pattern and trigger ventricular arrhythmias [16–18].

The SCN5A gene in BrS regulates cardiac sodium channels in an anomalous way, this effect can worsen with anti-malarial agents. Quinine works by blocking Na2 +channels, where further Ito activity is generated, ICaL activity decreases and INa activity is reduced. Thus, quinine leads to early depolarization or delayed depolarization, which can exacerbate BrS arrhythmias [15, 19].

Currently, the recommended therapy for malaria in this situation is the introduction of DHP. In addition to its anti-malarial effects, Jeong et al. reported antiarrhythmic effects on Ito and Iks channels in a mouse model [20]. It turns out, studies by Borsini et al*.* observed that DHP and primaquine also have ion blocking effects of Nav1.5, Kv4 and Kv11.1 channel that will develops QT interval prolongation, which induce a greater Ventricular Tachycardia/Fibrillation (VT/VF) [7, 21]. Therefore, DHP is assumed to suppress the occurrence of tachyarrhythmias in patients with BrS only for a short period, but along the way, it also provokes QT interval prolongation. It has been well reported that prolongation of the QT interval triggers ventricular arrhythmias, a known cause of sudden cardiac death.

Artemisinin-based combinations are unique in their own way, it has proarrhythmic and antiarrhythmic effects [22, 23]. The American Heart Association has summarized common drugs such as antiarrhytmic, tricyclic antidepressants, anesthetics/analgesic can trigger BrS, with ajmaline that most likely induced BrS [18].

The current consensus on the management of BrS states that ICD implantation is recommended as definitive therapy for patients with a documented history and risk of cardiac arrest or spontaneous tachycardia [24]. The standard follow-up methods that can be used in addition to clinical improvement include monitoring for arrhythmic symptoms and standard ECG or Holter monitoring [25, 26].

## Conclusion

In countries where malaria is endemic and prevalent, clinicians should be particularly vigilant for cardiac adverse events in patients with malaria, particularly in young Asian men. Therefore, these findings should help clinicians identify this unusual presentation meticulously while optimizing patient outcomes.

## Data Availability

The data that support the findings of this case report were taken from the medical records of Dr. Wahidin Sudirohusodo Hospital and are available from the corresponding author upon reasonable request. No datasets were generated or analysed during the current study.

## Abbreviations

BrS: Brugada syndrome
CVD: Cardiovascular Disease
DHP: Dihydroartemisinin-piperaquine
Ito: Transient outward potassium current
Ikr: Potassium rapid delayed rectifier current
Iks: Potassium slow delayed rectifier current
ICaL: Inward L type calcium current
Ina: Sodium current
ECG: Electrocardiogram
ICD: Implantable cardioverter Defibrillator
VT: Ventricular Tachycardia
VF: Ventricular Fibrillation
ACT: Artemisinin-based combination therapy
